# Exploring the potential for foreign-trained dentists to address workforce shortages and improve access to dental care for vulnerable populations in the United States: a case study from Washington State

**DOI:** 10.1186/1472-6963-10-336

**Published:** 2010-12-10

**Authors:** Naseem Bazargan, Donald L Chi, Peter Milgrom

**Affiliations:** 1 University of Washington School of Dentistry, Department of Dental Public Health Sciences, 1959 NE Pacific Street, Box 357475, Seattle, WA, 98195, USA

## Abstract

**Background:**

To address dental workforce shortages in underserved areas in the United States, some States have enacted legislation to make it easier for foreign dental school graduates to become licensed dentists. However, the extent to which foreign dental school graduates will solve the problem of dental workforce shortages is poorly understood. Furthermore, the potential impact that foreign-trained dentists have on improving access to dental care for vulnerable patients living in dental Health Professional Shortage Areas (HPSAs) and those enrolled in public insurance programs, such as Medicaid, is unknown. The objective of this paper is to provide a preliminary understanding of the practice behaviors of foreign-trained dentists. The authors used Washington State as a case study to identify the potential impact foreign dental school graduates have on improving access to dental care for vulnerable populations. The following hypotheses were tested: a) among all newly licensed dentists, foreign-trained dentists are more likely to participate in the Medicaid program than U.S.-trained dentists; and b) among newly licensed dentists who participated in the Medicaid program, foreign-trained dentists are more likely to practice in dental HPSAs than U.S.-trained dentists.

**Methods:**

The authors used dental license and Medicaid license data to compare the proportions of newly licensed, foreign- and U.S.-trained dentists who participated in the Medicaid program and the proportions that practiced in a dental HPSA.

**Results:**

Using bivariate analyses, the authors found that a significantly lower proportion of foreign-trained dentists participated in the Medicaid program than U.S.-trained dentists (12.9% and 22.8%, respectively; P = 0.011). Among newly licensed dentists who participated in the Medicaid program, there was no significant difference in the proportions of foreign- and U.S.-trained dentists who practiced in a dental HPSA (P = 0.683).

**Conclusions:**

Legislation that makes it easier for foreign-trained dentists to obtain licensure is unlikely to address dental workforce shortages or improve access to dental care for vulnerable populations in the United States. Licensing foreign dental school graduates in the United States also has ethical implications for the dental workforces in other countries.

## Background

Inadequate access to dental care services is a problem for vulnerable populations in the United States (U.S.), in part because there is a geographic maldistribution of dentists [1-3]. Over 49 million individuals in the U.S. reside in a dental Health Professional Shortage Area (HPSA) [4,5]. Dental HPSAs are federally-designated regions with an insufficient number of dentists to meet the needs of the local population. There are three types of dental HPSAs: i) geographic (e.g., areas in which the population to dentist ratio is greater than 5,000 to 1); ii) demographic (e.g., areas with large low-income, migrant, or Native American populations); and iii) facility (e.g., prisons) [6,7].

In response to the increased demand for dental care services in dental HPSAs, efforts have been directed at creating a network of safety-net dental clinics [8-10]. Each year, eight million patients access dental care in safety-net clinics, many of which are located in community health centers, regional hospitals, public schools, and dental schools [8,11,12]. Many of these patients are enrolled in state Medicaid programs, a public health insurance program for economically vulnerable children, adults, and families. However, efforts to expand the dental safety-net to cover more patients have led to modest improvements, leaving large proportions of vulnerable populations with inadequate access to dental services [8]. To address U.S. dental workforce shortages, a proposed solution is to grant dental licenses to foreign dental school graduates (or "foreign-trained dentists") [13,14]. This approach is based on existing models from medicine and nursing.

### Foreign Medical and Nursing School Graduates in the U.S. Healthcare System

Historically, the U.S. has relied heavily on foreign-trained health care professionals to compensate for workforce shortages. This trend has intensified over the last half century [15-18]. For example, a 2005 study reported that 25% of the U.S. physician workforce consists of foreign medical school graduates [16]. In fact, estimates suggest that one-fourth of all community health centers in the U.S. fill physician vacancies with foreign medical school graduates [18]. In nursing, 3.5% of the U.S. workforce in 2004 was comprised of foreign nursing school graduates [19,20].

### Little is Known about the Foreign-Trained Dentist Population in the U.S

In contrast to medicine and nursing, very little is known about the size and distribution of foreign-trained dentists in the U.S. dental workforce. A 2007 study examined data from the National Board Dental Examinations (NBDE) to identify the number of foreign-trained dentists practicing in the U.S. [21]. The authors estimated that 17% of dentists who passed the NBDE Part II between 2002 and 2005 graduated from a non-U.S. dental school. Presumably, not all of these dentists went on to become licensed dentists. However, these estimates suggest that foreign-trained dentists comprise a significant proportion of the U.S. dental workforce. As such, foreign-trained dentists could have an important role in improving access to dental care for vulnerable populations, especially for patients living in dental HPSAs or those enrolled in Medicaid, either by starting private practice dental offices in these areas or filling vacancies in safety-net clinics. However, there is no empirical evidence to support this statement.

### Dental Licensure in Washington State

Prior to 1985, it was difficult for foreign-trained dentists to obtain a dental license in Washington State. Subsequently, legislation was enacted to allow foreign-trained dentists to apply for licensure by examination after successfully passing NBDE Parts I and II, completing a Commission on Dental Accreditation (CODA) approved two-year pre- or post-doctoral dental education program, and passing a clinical licensing exam [22]. Washington also grants dental licenses by endorsement for dentists previously licensed in another state, as long as the applicant is a graduate of a CODA or Washington State Dental Quality Assurance Commission-approved dental school.

The University of Washington (UW) is the only accredited dental school in a five-state area comprised of Washington, Wyoming, Alaska, Montana, and Idaho. Unlike 32 of 58 accredited dental schools in the U.S., the UW has not offered a Program for Advanced Standing Students (PASS). PASS typically admits graduates of foreign dental schools to the second or third year of dental school. Furthermore, the UW is not one of the 15 dental schools in the U.S. that has an International Dentist Program (IDP), which enables foreign-trained dentists to enroll as first year or advanced standing dental students [23,24]. Plans are currently in place to inaugurate an IDP at the UW in 2011. A small number of foreign-trained dentists are admitted to the UW's post-graduate dental residency programs (e.g., oral medicine, pediatric dentistry, periodontics).

### Problem Statement and Study Hypotheses

Washington, similar to most other states, lacks a formal tracking system to identify where dentists were educated, where they currently practice, and what populations they serve. As a result, the impact that foreign-trained dentists have on improving access to dental care for patients living in dental HPSAs and other vulnerable populations is unknown.

Our objective in this paper is to provide a preliminary understanding of the practice behaviors of foreign-trained dentists. We used dental license application and Medicaid provider license data from Washington State to test the following hypotheses:

• Among all newly licensed dentists, foreign-trained dentists are more likely to participate in the Medicaid program than U.S.-trained dentists; and

• Among newly licensed dentists who participate in the Medicaid program, foreign-trained dentists are more likely to practice in dental HPSAs than U.S.-trained dentists.

## Methods

### Study Setting

There are 6.5 million people in Washington State and about one-half of the population lives in the metropolitan areas of Seattle, Tacoma, and Bellevue [25]. In 2009, 33 areas in Washington were designated as dental HPSAs [26]. Of the 39 counties in Washington, 85% had a partial dental HPSA designation and 67% were designated as entire county dental HPSAs [26]. One-half of Washington State's population is enrolled in Medicaid or has no dental insurance [27]. More specifically, 15% of children under age 17 had no dental insurance in 2003; 30% of adults ages 18-64 and 65% of those older than age 65 had no dental insurance in 2001 [28].

In 2006, there were 4,473 licensed dentists in Washington [28]. Lower-income rural and urban areas had a lower dentist-to-population ratio than higher-income areas [28,29]. There is also a wide range in dentist-to-population ratios across the state. For example, the dentist-to-population ratio in urban King County is 1:928, while rural Pend Oreille County has a ratio of 1:12,300 [28]. In December 2006, Skamania County had no dental Medicaid providers [28]. Additionally, it is estimated that 50% of all full-time general dentists in the State plan to retire by the year 2013, which may exacerbate workforce shortages, particularly in rural areas where older dentists typically practice [30].

### Data Source and Study Population

We obtained dental license application and Medicaid provider license data from the Washington State Department of Health and the Washington State Department of Social and Health Services, respectively. Our study sample consisted of all foreign- and U.S.-trained dentists in Washington State who were newly licensed between September 1, 2006 and September 30, 2008 (N = 688).

### Study Variables

We abstracted descriptive data from each dental license application. Variables included dental license number, date of birth (used to calculate age), sex, dental school training (foreign vs. U.S.), and method of licensing (examination vs. endorsement). All dentists in Washington State have a unique dental license number, which is necessary to apply for a Medicaid provider number. We used the dental license number to determine whether the dentist had a Medicaid provider number, which served as a proxy for participation in the Medicaid program. It was possible to match these data for all dentists in our study who had received a Medicaid provider number within one year of receiving a Washington State dental license. For dentists with a Medicaid provider number we used publicly-available dental practice zip codes to create two practice-level variables:

• whether the practice was in a dental HPSA as designated by the Health Resource and Services Administration [31]; and

• the rurality of the practice (based on Rural-Urban Commuting Area [RUCA] Codes with large and small isolated rural towns classified as "rural" and urban and suburban towns classified as "urban").

### Data Analysis

After we generated univariate descriptive statistics, individual- and practice-level characteristics for foreign- and U.S.-trained dentists were compared using chi-square and t-tests (α = 0.05). The chi-square test was used to evaluate our study hypotheses that foreign-trained dentists are more likely to participate in the Medicaid program and more likely to practice in a dental HPSA than U.S.-trained dentists. All analyses were completed with PASW Statistics for Windows Version 17.0 (formerly called SPSS).

## Results

### Individual- and Practice-Level Characteristics (Table 1)

**Table 1:** Demographic Characteristics of All Newly Licensed Dentists, U.S.-Trained Dentists, and Foreign-Trained Dentists in Washington State

Measure	All Dentists	U.S.- Trained Dentists	Foreign-Trained Dentists	Significance Testing between U.S.-Trained and Foreign-Trained Dentists (α = 0.05)
Individual-Level Characteristics*				

Dental school training				n/a
Foreign-trained dentist	139 (20.2)	n/a	n/a	
U.S.-trained dentist	549 (79.8)	n/a	n/a	

Age (years)				P = 0.865
Mean ± Standard Deviation	34.7 ± 8.4	34.7 ± 9.0	34.8 ± 5.1	

Sex, n (%)				P < 0.0001
Female	258 (37.5)	177 (32.2)	81 (58.3)	

Method of licensing, n (%)				P < 0.0001
Examination	443 (69.5)	327 (65.5)	116 (84.1)	
Endorsement	194 (30.5)	172 (34.5)	22 (15.9)	

Participated in the Medicaid				P = 0.011
Program				
Yes	143 (20.8)	125 (22.8)	18 (12.9)	

Practice-Level Characteristics**				

Practice is in a dental Health				P = 0.726
Professional Shortage Area				
Yes	66 (46.2)	57 (45.6)	9 (50.0)	

Rurality of practice				P = 0.683
Urban	114 (79.7)	99 (79.2)	15 (83.3)	
Rural	29 (20.3)	26 (20.8)	3 (16.7)	

* Includes all dentists in the study population (N = 688)

**Includes only dentists in the study population with a Washington State Medicaid provider number (n = 143)

There were 688 newly licensed dentists in Washington State between September 1, 2006 and September 30, 2008. About 20% of dentists were foreign-trained (n = 139). One-in-four graduated from a dental school in India; 24.5% from Asia (China, Taiwan, South Korea, Thailand, Philippines); 16.5% from Central or South America; 10.8% from the Middle East; 10.1% from Europe; 7.9% from Canada; and 5.6% from another area.

There was no significant difference in the mean age between foreign- and U.S.-trained dentists (P = 0.865). Significantly larger proportions of foreign-trained dentists were females than were U.S.-trained dentists (58.3% and 32.2%, respectively; P < 0.0001). Most dentists were licensed by examination, with significantly larger proportions of foreign-trained dentists being licensed by examination than U.S.-trained dentists (84.1% and 65.5%, respectively; P < 0.0001).

One-in-five newly licensed dentists participated in the Medicaid program (n = 143). Over 90% of these dentists had a Medicaid provider number within nine months of being licensed. A significantly lower proportion of foreign-trained dentists participated in the Medicaid program than U.S.-trained dentists (12.9% and 22.8%, respectively; P = 0.011). Among dentists who participated in the Medicaid program, 46.2% of dentists practiced in a dental HPSA and 79.7% were in an urban area. There were no significant differences in practice-level characteristics, such as the dentist's practice being located in a dental HPSA or rurality, between foreign- and U.S.-trained dentists.

## Discussion

This is the first study, to our knowledge, that examined the potential role that foreign-trained dentists can have on addressing dental workforce shortages and improving access to dental care for vulnerable populations. We used Washington State as a case study to test the hypotheses that compared to U.S.-trained dentists, foreign-trained dentists are more likely to participate in the Medicaid program and more likely to practice in a dental HPSA. Based on our findings, we arrived at two preliminary conclusions. First, significantly lower proportions of newly licensed, foreign-trained dentists participated in the Medicaid program than newly licensed, U.S.-trained dentists. Second, among newly licensed dentists who participated in the Medicaid program, there was no significant difference in the proportions of foreign- and U.S.-trained dentists practicing in a dental HPSA.

A possible explanation for our findings relates to dental school debt. School debt influences recent graduates' practice behaviors [32]. A 2004 study conducted by the American Dental Education Association found that 90% of dental school seniors graduated with a mean student debt of $135,721 [33]. In the early 1970s, the Institute of Medicine found that dental education subsidies were unnecessary, as the rate of return on dental education was large and subsidies did not yield more dentists who served the poor [34]. Consequently, the cost of dental education has continued to increase, leading to a concomitant rise in indebtedness [33,35]. It is unknown if foreign-trained dentists have greater student debt than U.S.-trained dentists.

After becoming licensed, one practice option for dentists is to work at a community health center, many of which serve Medicaid-enrolled patients and are located in dental HPSAs. Our findings suggest that newly licensed, foreign-trained dentists are not more likely to work in community health centers than U.S.-trained dentists. The average income of a community health center dentist is $73,000 less than that of a private practice dentist [36,37]. This income differential may drive many foreign-trained dentists to the second practice option, which is to open their own private practice office or become an associate dentist in an established practice. It is common for experienced dentists to employ associates to grow their practices. Associate dentists may or may not become Medicaid providers, depending on the type of practice they join. Anecdotal evidence suggests that established dental practices with the capacity to hire an associate dentist are less likely to accept Medicaid patients given the busyness of the practice, which could explain why foreign-trained dentists are less likely to see Medicaid patients and not more likely to practice in a dental HPSA. In addition, 58.3% of foreign-trained dentists in our study were female. Previous work based on data from Washington State suggests that female dentists have different practice patterns from male dentists [38]. As such, sex may confound the relationship between where a dentist was trained (U.S. versus foreign dental school) and the likelihood of treating vulnerable populations, a hypothesis worthy of further investigation.

Another explanation for our findings is that foreign-trained and U.S.-trained dentists, broadly speaking, may not differ on characteristics related to willingness to treat the underserved. It is a commonly held assumption that licensing foreign-trained health care professionals is the solution to workforce shortages [39,40]. This may not be the case. A 2010 study found that efforts in Washington State to recruit foreign medical school graduates to medical HPSAs are effective at initially recruiting but less effective at retaining physicians in underserved areas [41]. As in medicine and nursing, it is likely that foreign-trained dentists will continue to be an important part of the dental workforce in the future. However, long-term solutions to the maldistribution of dentists that involve foreign-trained dentists need to ensure that dentists locate to and remain in areas with the greatest need. In addition, policies that encourage foreign-trained dentists potentially result in "brain drain" abroad and reduce the capacity of foreign healthcare systems to serve their populations, which introduces ethical concerns [15]. States must weigh these ethical considerations when developing licensing policies.

One-fifth of newly licensed dentists on our study were foreign-trained, suggesting that current policies in Washington State are not as restrictive as they were prior to 1985, when licensure rules changed. While other States such as Maryland, Massachusetts, and California have recently implemented innovative programs that make it easier for foreign-trained dentists to obtain licensure [23], there are no published evaluation data on these programs, making it to difficult to compare findings. However, the proportion of newly licensed, foreign-trained dentists in Washington is slightly greater than the estimated 17% of dentists in the U.S. presumed to be foreign-trained between 2002 and 2005 [21].

It is worrisome that only 20% of all newly licensed dentists in our study participated in the Medicaid program, which is the only way a dentist can be reimbursed for providing dental care to Medicaid-enrolled patients. While it is possible that some of these dentists may have become Medicaid providers at a later time, this is unlikely. A 2001 study found that newly graduated dentists in Louisiana were more likely to be active Medicaid providers than established dentists, which suggests that Medicaid participation is highest during the earliest years of practice [42]. Our findings highlight a potential problem that the Washington Medicaid Program may have in recruiting new dentists to the program. Future efforts may need to be directed at introducing and promoting the Medicaid program to new dentists, especially to those who may be unfamiliar with Medicaid, and encouraging established dentists to participate in the program.

### Study Limitations

Our study has two limitations. First, having a Medicaid provider number does not ensure that the licensee actually saw any Medicaid-enrolled patients, nor does it provide information on participation intensity. Second, our data are limited in scope. A comprehensive data set would have allowed us to evaluate more complex models and to assess the impact of other factors (such as level of student debt, family expectations, beliefs about dental access problems) in comparing practice-related behaviors of foreign- and U.S.-trained dentists. We believe that all states should systematically collect data on newly licensed foreign- and U.S.-trained dentists. These data should be easily linkable to Medicaid claims data for individual providers so that state Medicaid programs can be evaluated and compared. Effective programs could then be identified and promulgated in other states. In addition, future research efforts should be directed at collecting qualitative data from newly licensed dentists to identify the factors associated with the decision to treat vulnerable populations.

## Conclusions

Our findings suggest that newly licensed, foreign-trained dentists in Washington State are not more likely than U.S.-trained dentists to participate in the Medicaid program. Among those who participate in the Medicaid program, we found no significant difference in the proportion of foreign- and U.S.-trained dentists who practice in dental HPSAs. Thus, while licensing foreign-trained dentists has the potential to address dental workforce shortage problems that disproportionately affect vulnerable populations, our preliminary findings in this case study fail to support this commonly held belief. In addition, licensing foreign-trained dentists has ethical implications in terms of the accompanying brain drain that impacts the nations in which these dentists were trained. Future research and policies should be aimed at understanding how licensure policies for foreign-trained dentists can help to reduce disparities in access to dental care for vulnerable populations in the U.S., identifying the behavioral factors that drive newly licensed, foreign-trained dentists to treat vulnerable populations, and elucidating the ethical implications of state dental workforce policies on the oral health of citizens in other countries.

## Abbreviations used

(CODA): Commission on Dental Accreditation; (HPSA): Health Professional Shortage Area; (NBDE): National Board Dental Examinations; (PASS): Program for Advanced Standing Students; (RUCA): Rural-Urban Commuting Area Codes; (UW): University of Washington

## Competing interests

The authors declare that they have no competing interests.

## Authors' contributions

NB obtained and cleaned the datasets. All authors participated in the design of the study, were involved in data analyses, helped to draft the manuscript, and read and approved the final manuscript.

## Authors' information

At the time of this study, NB was a graduate student in the School of Public Health at the University of Washington. DC is Assistant Professor, Department of Dental Public Health Sciences, School of Dentistry, University of Washington. PM is Professor, Department of Dental Public Health Sciences, School of Dentistry, University of Washington; and Director, Northwest Center to Reduce Oral Health Disparities, University of Washington.

## Pre-publication history

The pre-publication history for this paper can be accessed here:

http://www.biomedcentral.com/1472-6963/10/336/prepub

## References

[B1] Krause D Frate DA May WL Demographics and distribution of dentists in Mississippi: a dental work force study J Am Dent Assoc 2005 136 5 668 77 15966657 10.14219/jada.archive.2005.0241

[B2] Harrison JP Daniel RC Nemecek V The growing importance of dental services in rural America Health Care Manag (Frederick) 2007 26 1 34 42 17314625 10.1097/00126450-200701000-00005

[B3] Chi DL Momany ET Kuthy RA Chalmers JM Damiano PC Preventive dental utilization for Medicaid-enrolled children in Iowa identified with intellectual and/or developmental disability J Public Health Dent 2010 70 1 35 44 Epub 2009 Aug 20 10.1111/j.1752-7325.2009.00141.x 19694935 PMC3592375

[B4] Shortage Designation: HPSAs, MUAs & MUPs http://bhpr.hrsa.gov/shortage/Archived by WebCite^® ^at [http://www.webcitation.org/5rjztkcVY]

[B5] Ruddy G Health Centers' Role in Addressing the Oral Health Needs of the Medically Underserved 2007 Washington, DC: Georgetown University Medical Center

[B6] Find Shortage Areas: HPSA by State & County http://hpsafind.hrsa.gov/Archived by WebCite^® ^at [http://www.webcitation.org/5rk02u15c]

[B7] Health Professional Shortage Areas and Medically Underserved Areas http://www.doh.wa.gov/hsqa/ocrh/HPSA/hpsa1.htm#Am%20I%20in%20a%20Designated%20Area Archived by WebCite^® ^at [http://www.doh.wa.gov/hsqa/ocrh/HPSA/hpsa1.htm#Am%20I%20in%20a%20Designated%20Area]

[B8] Bailit H Beazoglou T Demby N McFarland J Robinson P Weaver R Dental safety net: Current capacity and potential for expansion J Am Dent Assoc 2006 137 6 807 15 16803811 10.14219/jada.archive.2006.0294

[B9] Fallon LF Jr Schmalzried HD Henry SE Valasek T Earlie-Royer RS Creating a regional dental center serving six rural county health districts J Public Health Manag Pract 2010 16 4 325 8 20520371 10.1097/PHH.0b013e3181947258

[B10] Riedy CA Ly KA Ybarra V Milgrom P An FQHC Research Network in Oral Health: enhancing the workforce and reducing disparities Public Health Rep 2007 122 5 592 601 17877306 10.1177/003335490712200506 PMC1936953

[B11] Albert DA McManus JM Mitchell DA Models for delivering school-based dental care J Sch Health 2005 75 5 157 61 15989084

[B12] Shue BK Le H The framework for patient care at California community health center dental clinics J Calif Dent Assoc 2009 37 5 319 28 19831005

[B13] Factors Contributing to Low Use of Dental Services by Low-Income Populations 2000 Washington, DC: United States General Accounting Office

[B14] Rosenblatt RA Andrilla CHA Curtin T Hart LG Shortages of Medical Personnel at Community Health Centers: Implications for Planned Expansion JAMA 2006 295 9 1042 9 10.1001/jama.295.9.1042 16507805

[B15] Hooper CR Adding insult to injury: the healthcare brain drain J Med Ethics 2008 34 9 684 7 10.1136/jme.2007.023143 18757641

[B16] Mullan F The metrics of the physician brain drain New Engl J Med 2005 353 17 1810 8 10.1056/NEJMsa050004 16251537

[B17] Mejia A Migration of physicians and nurses: a world wide picture Bulletin of the World Health Organization 2004 82 8 626 30 15457641 PMC2622933

[B18] Zerehi MR The Role of International Medical Graduates in the U.S. Physician Workforce. A Policy Monograph of the American College of Physicians 2005 Philadelphia: American College of Physicians

[B19] The Registered Nurse Population: National Sample Survey of Registered Nurses, Preliminary Findings 2004 Washington, D.C.: U.S. Department of Health & Human Services, Health Resources & Services Administration

[B20] Polsky D Sochalski J Aiken LH Cooper RA Medical migration to the U.S.: trends and impact LDI Issue Brief 2007 12 6 1 4 17557414

[B21] Sweis LE Guay AH Foreign-trained dentists licensed in the United States: Exploring their origins J Am Dent Assoc 2007 138 2 219 24 17272378 10.14219/jada.archive.2007.0140

[B22] Legislature WS WAC 246-817-160 Graduates of nonaccredited schools 1995

[B23] American Dental Association. Summary of State Educational Requirements for International Dentists 2007 Chicago: American Dental Association

[B24] Boorberg NB Schonwetter DJ Swain VL Advanced placement, qualifying, and degree completion programs for internationally trained dentists in Canada and the United States: An overview J Dent Educ 2009 73 3 399 425 19289729

[B25] Albrecht DE Population Brief: Trends in the Western U.S., The State of Washington 2008 Logan, UT: Western Rural Development Center

[B26] Olexa L A Guide to Federal Health Professional Shortage Area and Medically Underserved Area/Population Designations in Washington State. Identifying needs for Washington State residents, including Medicaid and Medicaid Eligible populations 2009 Olympia: Office of Community Health Systems. Washington State Department of Health

[B27] Foster H Millions in this state must do without vital dental coverage Seattle Post-Intelligencer 2004

[B28] Byrappagari D Alves-Dunkerson J Chamie C Peters R The Impact of Oral Disease on the Lives of Washingtonians - The Washington State Oral Disease Burden Document 2007 Olympia: Washington State Department of Health Report No.: DOH Pub No. 160-001

[B29] Warren RC Oral Health for All: Policy for Available, Accessible, and Acceptable Care 1999 Connecticut Washington, DC: Center for Policy Alternatives

[B30] Dental Workforce Study 2001 Seattle: University of Washington, Washington State Dental Association

[B31] About HRSA http://www.hrsa.gov/about/default.htm Archived by WebCite^® ^at [http://www.webcitation.org/5rk0DNpL2]

[B32] Walton JN Matthew IR Dumaresq C Sudmant W The burden of debt for Canadian dental students: Part 4. The influence of debt on program and career decisions Journal Can Dent Assoc 2006 72 10 913 17187704

[B33] Chmar JE Weaver RG Valachovic RW Survey of dental student financial assistance, 2003-04 J Dent Educ 2005 69 11 1278 92 16275691

[B34] Cost of Education in the Health Professions: Report of a Study, Part III 1974 Washington, D.C.: National Academy of Sciences

[B35] Pyle M Andrieu SC Chadwick DG Chmar JE Cole JR George MC Glickman GN Glover JF Goldberg JS Haden NK Hendricson WD Meyerowitz C Neumann L Tedesco LA Valochovic RW Weaver RG Winder RL Young SK Kalkwarf KL ADEA Commission on Change and Innovation in Dental Education The case for change in dental education J Dent Educ 2006 70 9 921 4 16954413

[B36] Occupational Employment and Wages, May 2008 http://www.bls.gov/oes/2008/may/oes291021.htm#nat Archived by WebCite^® ^at [http://www.webcitation.org/5rk0IoMA1]

[B37] Bolin KA Shulman JD Nationwide survey of work environment perceptions and dentists' salaries in community health centers J Am Dent Assoc 2005 136 2 214 20 15782528 10.14219/jada.archive.2005.0146

[B38] del Aguila MA Leggott PJ Robertson PB Porterfield DL Felber GD Practice patterns among male and female general dentists in a Washington State population J Am Dent Assoc 2005 136 6 790 6 16022046 10.14219/jada.archive.2005.0265

[B39] Lopez N Berthold P Transnational licensure: foreign dentists in America reclaim their profession through the Program for Advanced Standing Students (PASS) J Am Coll Dent 2003 70 1 15 7 12772768

[B40] Mick SS Lee SY Wodchis WP Variations in geographical distribution of foreign and domestically trained physicians in the United States: 'safety nets' or 'surplus exacerbation'? Soc Sci Med 2000 50 2 185 202 10.1016/S0277-9536(99)00183-5 10619689

[B41] Kahn TR Hagopian A Johnson K Retention of J-1 visa waiver program physicians in Washington State's health professional shortage areas Acad Med 2010 85 4 614 21 10.1097/ACM.0b013e3181d2ad1d 20354376

[B42] Shulman JD Ezemobi EO Sutherland JN Barsley R Louisiana dentists' attitudes toward the dental Medicaid program Pediatr Dent 2001 23 5 395 400 11699161

